# An additional human chromosome 21 causes suppression of neural fate of pluripotent mouse embryonic stem cells in a teratoma model

**DOI:** 10.1186/1471-213X-7-131

**Published:** 2007-11-29

**Authors:** Afua Mensah, Claire Mulligan, Jackie Linehan, Sandra Ruf, Aideen O'Doherty, Beata Grygalewicz, Janet Shipley, Juergen Groet, Victor Tybulewicz, Elizabeth Fisher, Sebastian Brandner, Dean Nizetic

**Affiliations:** 1 Centre for Haematology, Institute of Cell and Molecular Science, Barts & The London, Queen Mary's School of Medicine, University of London, 4 Newark Street, London E1 2AT, UK; 2 Department of Neurodegenerative Disease, Institute of Neurology, UCL, Queen Square, London WC1N 3BG, UK; 3 Division of Immune Cell Biology, MRC-National Institute for Medical Research, Mill Hill, London NW7 1AA, UK; 4 Male Urological Cancer Research Centre, The Institute of Cancer Research, Sutton, Surrey, SM2 5PT, UK

## Abstract

**Background:**

Down syndrome (DS), caused by trisomy of human chromosome 21 (HSA21), is the most common genetic cause of mental retardation in humans. Among complex phenotypes, it displays a number of neural pathologies including smaller brain size, reduced numbers of neurons, reduced dendritic spine density and plasticity, and early Alzheimer-like neurodegeneration. Mouse models for DS show behavioural and cognitive defects, synaptic plasticity defects, and reduced hippocampal and cerebellar neuron numbers. Early postnatal development of both human and mouse-model DS shows the reduced capability of neuronal precursor cells to generate neurons. The exact molecular cause of this reduction, and the role played by increased dosage of individual HSA21 genes, remain unknown.

**Results:**

We have subcutaneously injected mouse pluripotent ES cells containing a single freely segregating supernumerary human chromosome 21 (HSA21) into syngeneic mice, to generate transchromosomic teratomas. Transchromosomic cells and parental control cells were injected into opposite flanks of thirty mice in three independent experiments. Tumours were grown for 30 days, a time-span equivalent to combined intra-uterine, and early post-natal mouse development. When paired teratomas from the same animals were compared, transchromosomic tumours showed a three-fold lower percentage of neuroectodermal tissue, as well as significantly reduced mRNA levels for neuron specific (Tubb3) and glia specific (Gfap) genes, relative to euploid controls. Two thirds of transchromosomic tumours also showed a lack of PCR amplification with multiple primers specific for HSA21, which were present in the ES cells at the point of injection, thus restricting a commonly retained trisomy to less than a third of HSA21 genes.

**Conclusion:**

We demonstrate that a supernumerary chromosome 21 causes Inhibition of Neuroectodermal DIfferentiation (INDI) of pluripotent ES cells. The data suggest that trisomy of less than a third of HSA21 genes, in two chromosomal regions, might be sufficient to cause this effect.

## Background

Down's syndrome (DS), caused by the trisomy of human chromosome 21 (HSA21), [1] is a complex condition characterized by a plethora of phenotypic features, most striking of which are reduced neuron number and synaptic plasticity, early Alzheimer-like neurodegeneration, craniofacial dysmorphia, heart development defects, and powerful suppression of the incidence of most solid tumours [2,3]. In the first few months of life, DS babies display brachycephaly, microcephaly, delayed myelination, reduced growth of frontal lobes, a narrowing of the superior temporal gyrus, diminished size of the brainstem and cerebellum, and up to 50% reduction in numbers of cortical granular neurons [4-6]. The exact timing of onset of these changes is still unclear. In a limited study, neuronal progenitor cells from 3 foetal DS brains showed a reduced neuron number following further differentiation *in vitro*, compared to euploid foetal cells [7]. Also, DS foetal neural cells showed a reduced propensity for proliferation and survival *in vitro *compared to euploid controls [8]. Tumours of neural tissues, such as neuroblastomas, are comparatively very rarely observed in DS individuals [9,10].

Mouse models for DS display, among other features, behavioural and cognitive defects, synaptic plasticity defects and long term potentiation (LTP) deficits in the hippocampus, as well as reduced hippocampal and cerebellar neuron numbers [11-14]. The reduction of cerebellar granule neuron numbers in mouse models occurs in the first 10 days postnatally, due to a defective response to Sonic hedgehog (*Shh*) [15], secreted by an already reduced number of Purkinje neurons [11]. In the hippocampus, the exact timing of the onset of neuronal precursor cell reduction is less clear, but a reduced number of mitotically active granule neuron cell precursors is observed at day P6 [16]. At 4–6 months of age, mice undergo a neurodegenerative reduction of basal forebrain cholinergic neurons [17,18], further contributing to the reduction in granule cell neuron numbers [19].

Three quarters of human trisomy 21 concepti die in utero from developmental arrest [20], and phenotypic features of DS are retained even in mosaic DS subjects [21], as well as in transchromosomic DS mouse models where adult tissues retain <50% trisomic cells, having started from a fully trisomic conceptus [22,23]. This implies that many phenotypic features of DS must be determined by events occurring very early in development, but the exact nature of these early events, and the role played by increased dosage of individual HSA21 genes remain unknown. In an attempt to study the effects of trisomy 21 on the capacity of pluripotent embryonic stem (ES) cells to proliferate and differentiate *in vivo*, we report here the use of a mouse pluripotent embryonic stem (ES) cell line with a freely segregating HSA21 as a single supernumerary chromosome [24], in the generation of transchromosomic teratomas upon subcutaneous injections into syngeneic mice.

## Results

### Generation of teratomas

The cell line (47-1), generated by introduction of a single HSA21 into the mouse ES cell line D3, was found by PCR amplification of human specific markers to contain virtually all of the gene content of HSA21, and was shown not to contain DNA from any human chromosome other than HSA21 [24]. Transchromosomic 47-1 and control D3 cell lines were cultured under identical conditions and verified at the point of injection to be undifferentiated, having similar proliferation indices, and showing the presence of HSA21 in practically all 47-1 cells, and absence in D3 cells [see Additional files 1 &2]. Integrity of the retained HSA21 was verified by human specific PCR of 33 markers, and RT-PCR of 8 HSA21 genes. In 3 independent experiments (each starting from a new batch of frozen cells and following the entire experimental design as outlined in [see Additional file 1], a total of 30 mice were injected subcutaneously, each with 47-1 cells into the left flank, and D3 cells into the right flank. The resulting subcutaneous tumours were harvested 30 days later resulting in n = 24 mice - (left flanks, 47-1 injection point) and n = 21 mice (right flanks, D3 injection point). Sixteen animals developed tumours in both flanks. No significant difference in size/weight was found between 47-1 and D3 tumours (see Additional file 3). All tumours grew as solitary (single tumour per injection point) spheroid masses in the sub-cutaneous tissue, clearly separated from host tissues by an envelope of connective-like tissue.

### Histological analysis of the teratomas

Qualitative histological analysis of standard H&E stained tumour sections revealed a multitude of different cell types in all tumours. Scattered islands of many tissue types were observed including: keratinized squamous epithelium, ciliated epithelium, glandular epithelium, acini of salivary glands, smooth muscle, cartilage, bone, haematopoietic tissue, fat tissue and undifferentiated tissue. Many tumour slices lacked the presence of differentiated neuroectodermal tissue.

**Figure 1:**
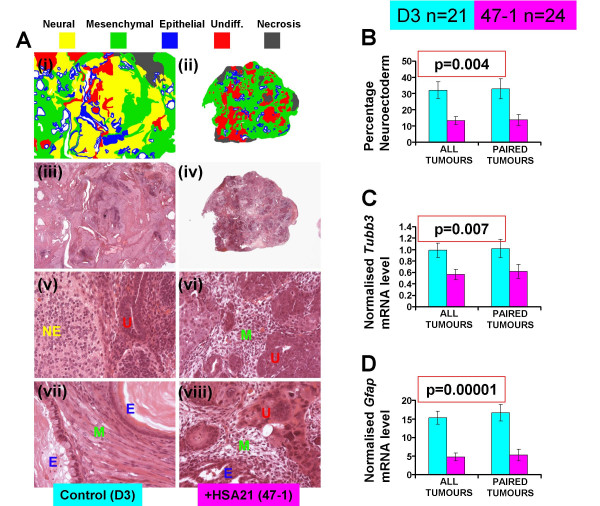
**Histology of wildtype and transchromosomic tumours and Inhibition of Neuroectodermal tissue DIfferetiation (INDI) by the supernumerary HSA21**. A freely segregating HSA21 has been introduced into the mouse ES cell line D3 to generate a transchromosomic line, 47-1, with an effective trisomy of the gene content of the entire HSA21. D3 and 47-1 cells were cultured in an undifferentiated state, under identical conditions, and verified at the point of injection to retain an apparently intact copy of HSA21 in practically all 47-1 cells and no D3 cells [see Additional file 1 & 2]. Thirty syngeneic mice were each injected subcutaneously with an identical inoculum of between 5–6.5 million 47-1 cells in the left flank, and the same number of D3 cells in the right flank. Resulting tumours were harvested 30 days post injection [experimental design see Additional file 1]. **A**, morphological classification of tissue types present in a typical pair of tumours which grew in the same mouse; D3 (left column, panels i, iii, v, vii) and 47-1 (right column, panels ii, iv, vi, viii). (i) and (ii) are representative sketches of (iii) and (iv), respectively. Note the absence of neuroectodermal tissue in the 47-1 tumour. **B**, the average neuroectoderm contents of all D3 and 47-1 tumours, and of D3 and 47-1 tumour pairs which grew in the same mouse (paired tumours, n = 16 pairs), as determined by analysis of H&E sections by a histopathologist blinded to the origin of the tumours. **C**, the average levels of Tubb3 mRNA expression (normalised to Gapdh mRNA levels) in all D3 and 47-1 tumours, and in D3 and 47-1 tumour pairs which grew in the same mouse, as determined by real-time quantitative RT-PCR; **D**, the average levels of Gfap mRNA expression (normalised to Gapdh mRNA levels) in all D3 and 47-1 tumours, and in D3 and 47-1 tumour pairs which grew in the same mouse, as determined by real-time quantitative RT-PCR.

**Figure 2:**
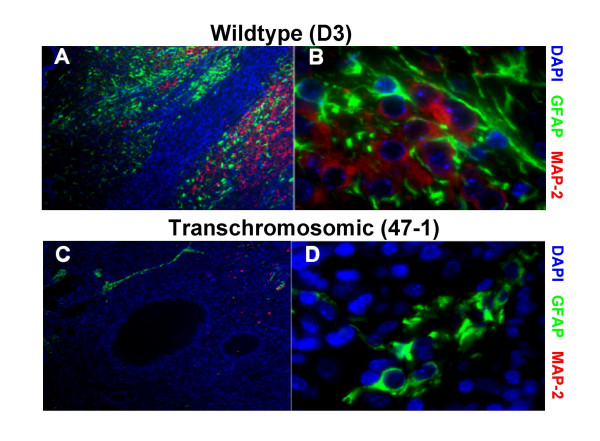
**Neurons and astroglia in a pair of wildtype and transchromosomic teratomas from the same animal**. The presence of the two main cell types of the differentiated neuroectoderm, neurons and astroglia, within the tumours was visualised by fluorescence immunocytochemistry. Paraffin-embedded sections of wildtype (D3) and transchromosomic (47-1) tumours were stained with antibodies specific for neuronal (MAP-2), and glial (GFAP) proteins. Transchromosomic tumours contained far fewer glia and neurons than wildtype tumours. **A**, D3 tumour at low magnification; **B**, high magnification photo of the same D3 tumour; **C**, 47-1 tumour at low magnification; **D**, high magnification photo of the same 47-1 tumour.

Tumour sections were analyzed quantitatively, by a histopathologist blinded to the tumour origin, for the presence and relative abundance (%) of four types of tissue: neuroectodermal, mesenchymal, epithelial and undifferentiated (Fig. 1A). Transchromosomic tumours showed an approximately three-fold lower mean percentage of neuroectodermal tissue, both when compared across all tumours (highly significant by two tailed t-test (p = 0.004), and when compared strictly within pairs of tumours which grew in the same animal (n = 16 pairs, paired t-test, p = 0.037), (Fig. 1B). The transchromosomic tumours also showed an almost two fold reduction of mRNA for mouse *Tubb3*, a neuron specific gene (Fig. 1C; t-test, p = 0.007, paired t-test p = 0.111 but non-overlapping standard error bars), and a three-fold reduction of mouse *Gfap *mRNA, a glia specific gene (Fig. 1D; t-test, p = 0.00001, paired t-test p = 0.003). The presence of both neurons and astroglia cells, respectively, within the tumours has been verified by immunofluorescence using antibodies against MAP2 and GFAP, which confirmed the observation of the reduced numbers of both types of cells in the transchromosomic tumours, compared to controls (Fig. 2).

### Analysis of a HSA21 region causing Inhibition of Neuroectodermal DIfferentiation (INDI)

**Figure 3:**
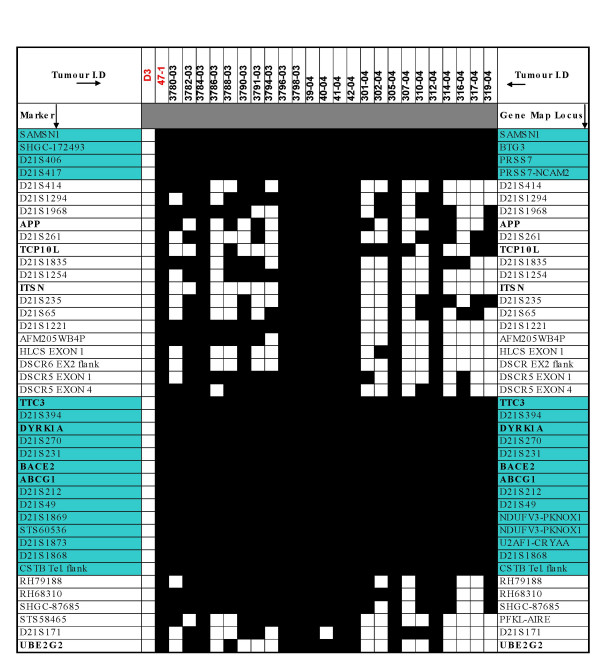
**Mapping of HSA21 deletions in DNA and RNA from transchromosomic tumours**. DNA and total RNA isolated from transchromosomic tumours were subjected to PCR or RT-PCR analyses, respectively, to determine the retention of each of 42 HSA21-specific markers in the transchromosome in the tumours. Shown on the right are the positions of markers relative to genes, and they correspond to the HSA21 STS markers analysed, listed on the left. cDNA markers are shown in bold text. PCR and RT-PCR analyses of DNA and RNA, respectively, isolated from D3 and 47-1 ES cell lines at the point of injection (first 2 columns, highlighted as red font) confirmed the full retention of HSA21 in 47-1 ES cells. No HSA21 markers were amplified in DNA or mRNA/cDNA from the D3 mouse control cell line. Markers in the 'INDI' regions that were commonly retained in all transchromosomic tumours are highlighted by green background. White box = no amplified product observed by agarose gel after 40 PCR cycles, black box = amplified product observed by agarose gel.

**Figure 4:**
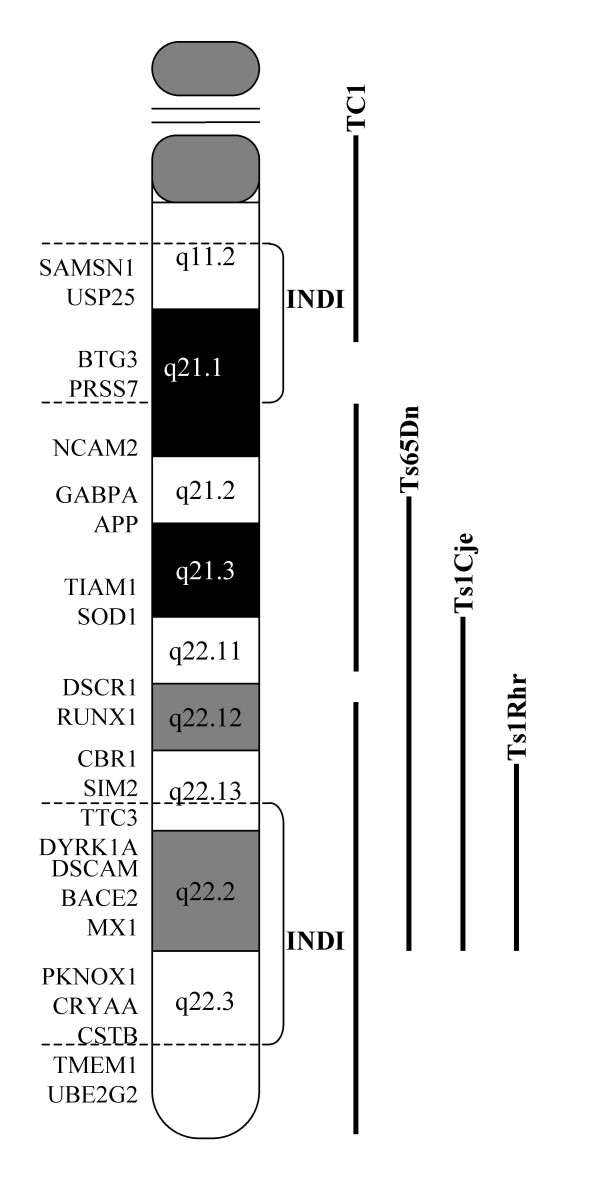
**A schematic diagram showing the boundaries of retained regions of HSA21 in transchromosomic teratomas, and their relationship to trisomic regions in mouse models of DS**. The two regions labelled "INDI" were commonly retained by all tumours. As there were sufficient numbers of cells of many different tissue types in all tumours, and multiple HSA21 PCR markers were negative in the deleted regions, deletions must have occurred before any differentiation of ES cells in the tumours took place. The regions of the HSA21 gene map which are trisomic in some of the current segmental trisomy mouse models are indicated on the right by vertical lines, for comparison.

Genomic DNA and total RNA of 47-1 tumours were analysed by PCR and RT-PCR, respectively, using the same battery of human sequence specific markers/genes that were verified to be present in the 47-1 cell line at the point of injection [see Additional file 1 and Fig. 3]. Two thirds of all 47-1 tumours showed a lack of amplification with multiple primers in the regions distal to PRSS7, proximal to TTC3, and distal to CSTB (Fig. 3). In the deleted segments, both genomic DNA and ubiquitously expressed cDNA markers failed to amplify. The percentage of cells within each tumour that might have lost the entire HSA21 were estimated by quantitative genomic DNA real-time PCR using a single marker within the commonly retained chromosomal segments normalized to a single mouse genomic DNA locus. An average level of 76% (+/- 8%) was obtained for retention of HSA21 in DNA material extracted from the 47-1 tumours. As there were sufficient numbers of cells of many different tissue types in all tumours, and multiple HSA21 PCR markers in the deleted regions gave no amplified product, deletions must have occurred before any differentiation of ES cells in the tumours took place. The presence or absence of deletions did not correlate with experimental chronology, or percentages of tissue types including neuroectoderm (not shown). This suggests that deletions most likely occurred during the initial cell divisions in undifferentiated ES cells at the very start of tumour growth. When only the deleted 47-1 tumours were compared to either all D3 tumours, or only their direct pairs, the statistically significantly reduced percentage of neuroectoderm, and levels of *Tubb3 *and *Gfap*, were observed, virtually identical to graphs shown in Fig 1. Regardless of the cause of the deletions, the data open themselves to interpretation that the segmental trisomy common to all tumours (Fig. 4), comprising less than a third of HSA21 gene complement, might be sufficient to cause a powerful Inhibition of Neuroectodermal DIfferentiation (INDI) in pluripotent ES cells *in vivo*.

## Discussion

The undifferentiated ES cell inoculates were allowed to develop for 30 days, equivalent to the time span of entire intrauterine, and 10 postnatal days of mouse development. This extended period encompasses the critical interval (P0–P6), in which the biggest difference in neuronal precursor cell proliferation was observed between mouse models of DS, and their euploid littermate controls [11,15,16]. The use of syngeneic inbred mouse strains also minimizes the individual variation, which is further reduced by the paired analysis of tumours which grow in the same recipient animal. Using this system, we have observed a powerful inhibition of neuroectodermal fate in transchromosomic +HSA21 containing pluripotent mouse ES cells, compared to their parental control (Fig 1A,B.). This conclusion is not without caveats: the inhibition of neural fate could be specific to the cell line used, or it could be related to the sheer presence of a supernumerary chromosome, not specific to HSA21. Though it is impossible to rule out these caveats at this stage, we believe on balance of probabilities, that this system shows a measurable phenotype with potential to map it to a segment of HSA21. The reduced Tubb3 levels (Fig 1C) clearly indicate a reduction in mature neuron numbers in trisomic teratomas, confirmed by neuron specific staining (Fig 2). The reduction in GFAP levels could be partly the result of reduced numbers of mature astrocytes, as well as reduced numbers of neuronal precursors, corresponding to GFAP+ cells found in the subgranular zone and hilus [25]. These GFAP+ precursors divide and give rise to immature neurons (DCX+PSA-NCAM+) [25]. Radial glial cells, which are the main source of precursors for neurogenesis in the dentate gyrus, also express GFAP [26]. These very cellular layers are the site of the biggest deficit in mitotic activity at postnatal day 6 in mouse models of DS [16].

Spontaneous deletions of the supernumerary HSA21 were observed in two thirds of transchromosomic tumours (Fig 3). As there were sufficient numbers of cells of many different tissue types in all tumours, and multiple HSA21 PCR reactions (including RT-PCR of ubiquitously expressed HSA21 genes) were negative in the deleted regions, deletions must have occurred before any differentiation of ES cells in the tumours took place, probably during the early cell divisions at the very start of tumour growth. Alternatively, a subset of cells with a major deletion might have been present within the inoculum of 47-1 cells; a less likely explanation, as 3 independent cultures gave rise to a similar proportion of tumours with, and without deletions. We cannot exclude the possibility that the inocula in all 3 experiments contained a mixture of two clones, the 47-1, and a segmental trisomy clone. However, the deleted segments also show subtle variations in the pattern of markers present, making this possibility less likely. Whether the deletions are caused by an instable DNA sequence element, and whether or not they provide a selective advantage to the proliferation of cells, remains to be investigated. Regardless of the cause of deletions within the tumours, they had no effect on the statistical significance of any differences shown in Fig. 1. Though the data are not conclusive on this point, it can be hypothesized that the segmental trisomy of two regions common to all tumours (Fig. 4), comprising less than a third of the HSA21 gene complement, could be sufficient to cause a powerful Inhibition of Neuroectodermal DIfferentiation (INDI) in pluripotent ES cells *in vivo*.

The telomeric INDI region partially overlaps with trisomic regions in mouse segmental trisomy models for DS, such as Ts65Dn and Ts1Cje (Fig. 4), which both have a reduced number of granular layer neurons in the cerebellum and hippocampus, compared to euploid littermates [11,16,27]. The overlap between the telomeric INDI segment and the 3 mouse models shown in Fig. 4. is restricted to 23 genes (based on comparisons, see refs [1,28]. The centromeric INDI region is less likely to play a causative role in INDI, as the degree of cerebellar neuron number reduction is similar between the Tc1 mouse model [23] which has this region in trisomy, and the Ts65Dn and Ts1Cje models that do not [1]. The exception are only 3 genes from the centromeric INDI region (BTG3, YG81 and PRSS7) which are deleted in the third chromosome of the Tc1 model [23], and therefore remain unchecked. The telomeric INDI segment of 23 genes overlapping with trisomic mouse models is fully contained within the Ts1Rhr, the 33 gene-trisomy mouse model, which does not show the reduction in cerebellar volume, or neuron density [28]. This mouse model also shows no hippocampal volume change, and no electrophysiological and behavioural defects associated with hippocampal functions, therefore suggesting that trisomy of this segment is not sufficient to cause the brain pathology in the mouse [28]. However, this 33 gene segment was found necessary for most of the cerebellar and hippocampal pathology of Ts65Dn model, as when its trisomy is reversed to disomy, the pathologies disappeared [28]. This segment also probably contains highly and bi-directionally dose sensitive genes regulating brain development, as a monosomy of this segment (mouse model Ms1RhR) produces striking changes in cerebellar and hippocampal volume and neuron density [28]. The data in our system could differ from those in the Ts1RhR model due to the human, rather than mouse, origin of the third chromosome. Interestingly, very recent data in human DS show that duplication of a 4.3 Mb segment, completely contained within the 33 gene equivalent segment in the mouse, is sufficient to cause (in three members of the same family) a range of DS phenotypes, including brachycephaly, intellectual disability, mental retardation, speech learning impairment, and a typical facial gestalt of DS [29]. Our data suggest that further studies of individual gene dosage effects within the INDI region could reveal major candidate contributors to DS-related hypo-cellularity of the CNS, and suppression of neuroblastomas.

## Conclusion

We demonstrate that a supernumerary chromosome 21 causes Inhibition of Neuroectodermal DIfferentiation (INDI) of pluripotent ES cells. The data open themselves to interpretation that the trisomy of less than a third of HSA21 genes, in two chromosomal regions, could be sufficient to cause this effect.

## Methods

General outline of the experimental design is summarized [see Additional file 1].

### ES cell lines and culture

The transchromosomic cell line 47-1, described in a previous publication [24], has been produced by tagging HSA21 with a Neomycin resistance marker, and introducing the tagged chromosome into a mouse embryonic stem cell line D3, using microcell mediated chromosome transfer. ES cells were grown on a layer of mitotically inactivated mouse embryonic fibroblasts (feeder cells), in medium supplemented with LIF (ESGRO). ES medium: DMEM, 15% FCS, 25,000 U Pen/Strep, L-Glutamine, non-essential amino acids, β-mercaptoethanol, 5 × 105 U/ml LIF. 47-1 ES cells were also grown in the presence of G418 (500 mg/ml), until one passage before they were harvested for injections. During this last passage the G418 was removed, so that the 47-1 and D3 cells had identical culturing conditions. At the point of injection, aliquots of both cell suspensions have been verified by phase contrast microscopy to contain morphologically undifferentiated cells, with similar proliferation indices, as measured by visualizing the cells in mitosis using a Phosphohistone H3-specific antibody [see Additional file 2]. One aliquot each of the 47-1 and D3 ES cells was cultured without feeders for one passage, and interphase nuclei were prepared for FISH using a HSA21-specific centromeric probe pZ21A, and standard protocol for interphase nuclei FISH [30]. HSA21 retention was confirmed in 96/100 examined 47-1 cells, and 0/100 D3 [see Additional file 2].

### Injections and tumour utilization

The use of mice was approved by the institutional ethics committee, and by the Home Office project licence (PPL 70/5714). ES cells were trypsinised, washed twice in sterile 1× PBS and resuspended at a density of 6.5 × 106 cells/200 μl (experiment 1), or 5 × 106 cells/200 μl (experiments 2 and 3) immediately before injections. Equal numbers of cells in 200 μl of D3 and 47-1 ES cell suspensions were subcutaneously injected into the right and left flanks respectively of each of a total of 30 male 129/Sv mice. Mice were monitored every two days for tumour growth (assessed by palpation and measurement with callipers). At 30 days post-injection, mice were sacrificed and tumours removed. Each tumour was cut in half. One half was placed in 10% buffered formalin and the other half snap-frozen in liquid nitrogen. Snap-frozen tumour halves were ground into powder before extracting total RNA and DNA. RNA extraction was carried out using RNABee (Biogenesis) in accordance with the manufacturer's protocol. DNA was extracted using 5% Chelex (Sigma) suspension.

### Histopathological analysis

Paraffin-embedded blocks of each tumour were cut in 3 μm thick sections and stained with H&E using standard protocols. Sections were analysed by a histopathologist blinded to the origin of the tumours, for the relative abundance of four types of tissue: neuroectodermal, mesenchymal, epithelial and undifferentiated, expressed as the percentage of the total tumour slice, as demonstrated with an example for a pair of tumours in Fig. 1A.

### Immunofluorescent staining

Cytospins of D3 and 47-1 ES cell suspensions remaining from injections in experiment 1 were fixed with 2% PFA and stained with antibody specific for Phospho-Histone H3 (1:1000) to determine ES cell viability at the point of injection. Alexa Fluor 594 (Molecular probes) was used a secondary antibody (1:800). Paraffin sections of tumours were stained with anti-GFAP rabbit polyclonal (DAKO,1:1000) and anti-MAP-2 monoclonal (Chemicon,1:500) antibodies, and visualised with Alexa Fluors 488 and 594 respectively (Molecular probes, 1:800). Immunofluorescently stained cells and tumour sections were viewed using a Zeiss Axioskop and the Quips Smartcapture Imaging Software (Vysis).

### Quantitative RT-PCR for tissue specific markers

Expression levels of mouse Tubb3 and mouse Gfap mRNAs were assessed by real-time PCR in an ABI 7700 system, using SYBR Green as the reporter dye and mouse *Gapdh *as the endogenous normalizing control. Primers for real-time PCR were designed using Primer Express software (ABI). Tubb3 primers: 300 nM FWD/300 nM REV (GCTGTCCGCCTGCCTTTT/GACCTCCCAGAACTTGGCC). Gfap primers: 900 nM FWD/300 nM REV (GAAAACCGCATCACCATTCC/TCGGATCTGGAGGTTGGAGA). *Gapdh *primers: 300 nM FWD/50 nM REV (CCAGAAGACTGTGGATGGC/TGAGCTTCCCGTTCAGCTC). Normalized mRNA levels were calculated using the standard curve method.

### Assessment of integrity of HSA21 in the transchromosomic tumours

For mapping of the HSA21 retention in 47-1 tumours, STS primer sequences were obtained from the UniSTS database [31]. The ubiquitous expression of HSA21 cDNA markers was determined from assessment of the Unigene database [32], and data from expression catalogue studies of HSA21 genes [33,34]. DNA and cDNA primers were designed to be human specific in at least two of the most 3' bases. The exact sequences and conditions for each primer set are available on request.

## Authors' contributions

AM carried out cell culturing, tumour monitoring and dissection, PCR and immunostainings, and most of the analysis. CM and JG helped with retention and other PCR results, interpretation, statistical analysis and manuscript preparation. JL prepared histological sections of tumours for analysis, SR and AOD established the initial cultures and analysis of ES cells, BG and JS organized and checked the FISH analysis of transchromosomic ES cells and controls, VT and EF established the transchromosomic system and provided the cell lines, SB carried out the blind analysis of H&E sections of tumours, DN helped with tumour dissection, designed and coordinated the study, and wrote the manuscript.

## Supplementary Material

Additional file 1 **Procedure for generating wildtype and transchromosomic teratomas and the subsequent analysis of these tumours**. The transchromosomic ES cell line used, 47-1, is one of a panel of 21 transchromosomic ES cells generated by Hernandez et al (1999). The 47-1 cell line contains an entire, single, freely segregating HSA21 on the background of a normal mouse genome. Click here for file

Additional file 2 **FISH analysis of HSA21 retention in 47-1 ES cells and Phospho-Histone H3 analysis of proliferation rates of 47-1 and D3 ES cells**. **A**, 47-1 and D3 ES cells were stained with an HSA21-specific centromeric probe, pZ21A; **(i) **47-1 cells showed specific signal with the probe in 96/100 cells. **(ii) **No signals were detected in D3 ES cells. **B**, Phospho-Histone H3 staining to verify the presence of mitotic cells in **(i) **47-1 and **(ii) **D3 ES cell suspensions from the point of injection; D3 and 47-1 cells were shown have similar proliferation indices (as determined by a 2-tailed Student's t-test) Click here for file

Additional file 3 **Teratoma weights**. Weights of 47-1 tumours and their D3 counterparts (where present) which grew in the same injected animal. Click here for file
