# Supplementary material for: An additional human chromosome 21 causes suppression of neural fate of pluripotent mouse embryonic stem cells in a teratoma model
Source: BMC Dev Biol. 2007 Nov 29;7:131. doi: 10.1186/1471-213X-7-131 (PMC2211317; doi:10.1186/1471-213X-7-131)
Supplement: Additional file 1 — Procedure for generating wildtype and transchromosomic teratomas and the subsequent analysis of these tumours. The transchromosomic ES cell line used, 47-1, is one of a panel of 21 transchromosomic ES cells generated by Hernandez et al (1999). The 47-1 cell line contains an entire, single, freely segregating HSA21 on the background of a normal mouse genome. [file 1471-213X-7-131-S1.ppt]

## Slide 1
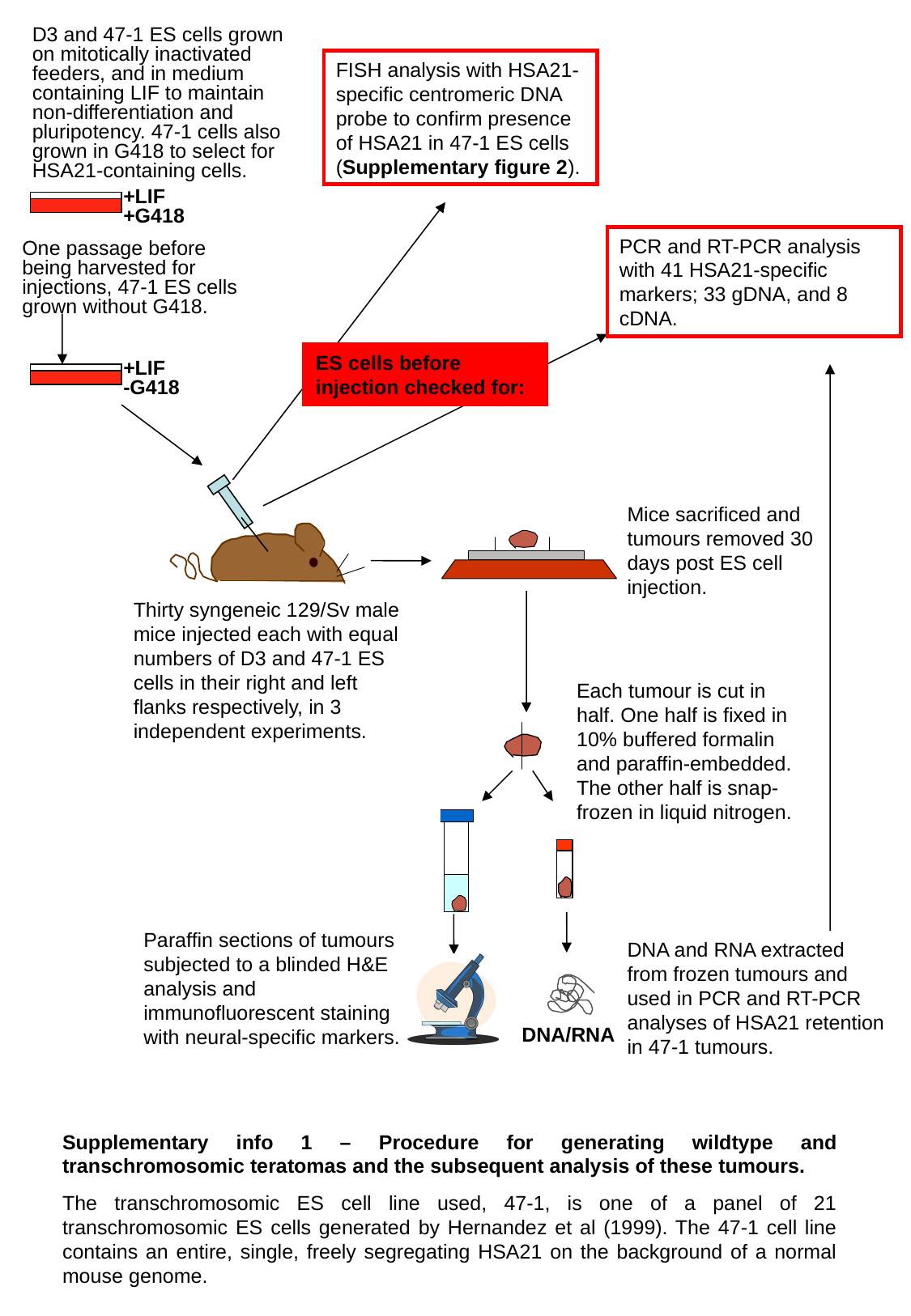

D3 and 47-1 ES cells grown on mitotically inactivated feeders, and in medium containing LIF to maintain non-differentiation and pluripotency. 47-1 cells also grown in G418 to select for HSA21-containing cells.
FISH analysis with HSA21-specific centromeric DNA probe to confirm presence of HSA21 in 47-1 ES cells (Supplementary figure 2).
+LIF
+G418
PCR and RT-PCR analysis with 41 HSA21-specific markers; 33 gDNA, and 8 cDNA.
One passage before being harvested for injections, 47-1 ES cells grown without G418.
ES cells before injection checked for:
+LIF
-G418
Mice sacrificed and tumours removed 30 days post ES cell injection.
Thirty syngeneic 129/Sv male mice injected each with equal numbers of D3 and 47-1 ES cells in their right and left flanks respectively, in 3 independent experiments.
Each tumour is cut in half. One half is fixed in 10% buffered formalin and paraffin-embedded. The other half is snap-frozen in liquid nitrogen.
Paraffin sections of tumours subjected to a blinded H&E analysis and immunofluorescent staining with neural-specific markers.
DNA and RNA extracted from frozen tumours and used in PCR and RT-PCR analyses of HSA21 retention in 47-1 tumours.
DNA/RNA
Supplementary info 1 – Procedure for generating wildtype and transchromosomic teratomas and the subsequent analysis of these tumours.
The transchromosomic ES cell line used, 47-1, is one of a panel of 21 transchromosomic ES cells generated by Hernandez et al (1999). The 47-1 cell line contains an entire, single, freely segregating HSA21 on the background of a normal mouse genome.
