# Supplementary figures and images for: An additional human chromosome 21 causes suppression of neural fate of pluripotent mouse embryonic stem cells in a teratoma model
Source: BMC Dev Biol. 2007 Nov 29;7:131. doi: 10.1186/1471-213X-7-131 (PMC2211317; doi:10.1186/1471-213X-7-131)

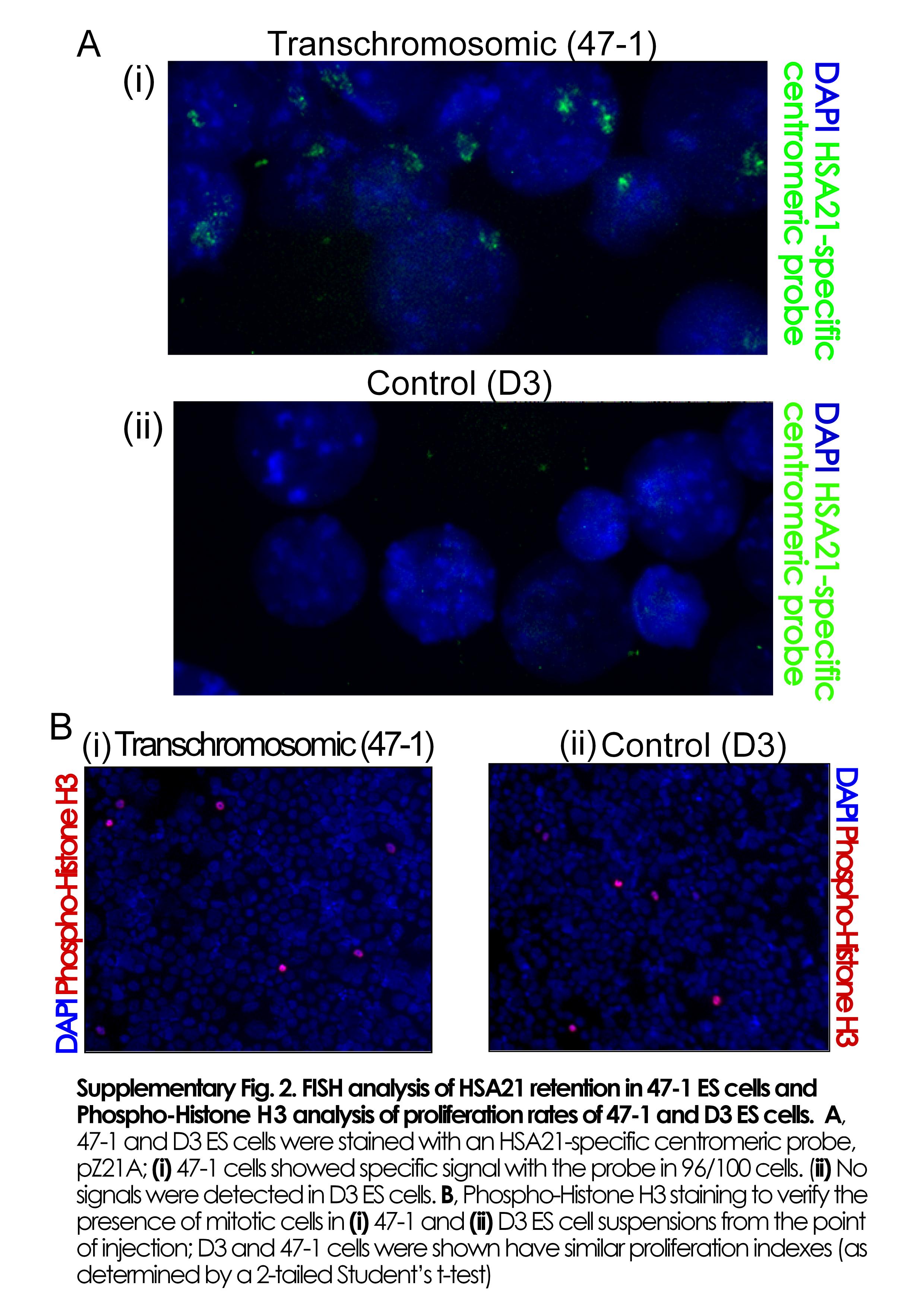

Supplement: Additional file 2 — FISH analysis of HSA21 retention in 47-1 ES cells and Phospho-Histone H3 analysis of proliferation rates of 47-1 and D3 ES cells. A, 47-1 and D3 ES cells were stained with an HSA21-specific centromeric probe, pZ21A; (i) 47-1 cells showed specific signal with the probe in 96/100 cells. (ii) No signals were detected in D3 ES cells. B, Phospho-Histone H3 staining to verify the presence of mitotic cells in (i) 47-1 and (ii) D3 ES cell suspensions from the point of injection; D3 and 47-1 cells were shown have similar proliferation indices (as determined by a 2-tailed Student's t-test) [file 1471-213X-7-131-S2.jpeg]
