# Supplementary material for: An additional human chromosome 21 causes suppression of neural fate of pluripotent mouse embryonic stem cells in a teratoma model
Source: BMC Dev Biol. 2007 Nov 29;7:131. doi: 10.1186/1471-213X-7-131 (PMC2211317; doi:10.1186/1471-213X-7-131)
Supplement: Supplementary file 3 [file 1471-213X-7-131-S3.doc]

47-1 tumc	weight (g)	D3 weight (g)
**3780-03**	0.34	absent
**3782-03**	0.52	2.75
**3784-03**	3.66	1.37
**3786-03**	0.11	1.09
**3788-03**	2.61	1.67
**3790-03**	0.54	1.2
**3791-03**	0.1	2.08
**3794-03**	0.06	absent
**3796-03**	0.39	absent
**3798-03**	5.42	0.02
**39-04**	0.03	0.26
**40-04**	0.05	2.59
**41-04**	2.93	0.10
**42-04**	4.18	0.06
**301-04**	0.90	absent
**302-04**	0.10	absent
**305-04**	0.90	0.10
**307-04**	0.1	absent
**310-04**	6.8	absent
**312-04**	4.30	0.3
**314-04**	4.5	0.3
**316-04**	0.2	absent
**317-04**	0.1	1.5
**319-04**	0.1	0.1
